# Mannose Inhibits NSCLC Growth and Inflammatory Microenvironment by Regulating Gut Microbiota and Targeting OGT/hnRNP R/JUN/IL-8 Axis

**DOI:** 10.7150/ijbs.107256

**Published:** 2025-01-27

**Authors:** Haoyi Jin, Huanghe He, Jijia Li, Xi Liu, Qi Cai, Jiang Shi, Zhexue Hao, Jianxing He

**Affiliations:** 1 Department of Thoracic Surgery and Oncology, the First Affiliated Hospital of Guangzhou Medical University, State Key Laboratory of Respiratory Disease & National Clinical Research Center for Respiratory Disease, Guangzhou 510120, China.; 2 Department of Thoracic Surgery, Cancer Hospital of China Medical University, Liaoning Cancer Hospital and Institute, Shenyang 110042, Liaoning, China.; 3 Department of Urology, Cancer Hospital of China Medical University, Liaoning Cancer Hospital and Institute, Shenyang 110042, Liaoning, China.

**Keywords:** non-small cell lung cancer, mannose, inflammatory microenvironment, gut microbiota, O-GlcNAc glycosylation

## Abstract

Recent studies have reported direct antitumor effects of mannose, a natural six-carbon monosaccharide, in the treatment of cancer. Herein, we utilized cancer cell lines, animal models, organoids and experimental techniques such as multi-omics and cellular experiments to investigate the regulatory effects of mannose on NSCLC growth and the inflammatory microenvironment. We demonstrated that mannose can inhibit cancer cell growth, inflammatory cell infiltration and inflammatory cytokine expression in NSCLC tissue, and enhance the antitumor efficacy of immune checkpoint inhibitor both *in vitro* and *in vivo*. Orally administered mannose increased the proportion of probiotics in the gut microbiota, the abundance of anti-inflammatory and antitumor metabolites in the blood and feces of NSCLC-bearing mice. In NSCLC cells, mannose reduced JUN mRNA stability and subsequent IL-8 transcription of NSCLC cells by directly targeting OGT to suppress the O-GlcNAc glycosylation of hnRNP R, which bound and stabilized JUN mRNA in an O-GlcNAc glycosylation dependent manner. Taken together, our study demonstrated that mannose can suppress NSCLC by inhibiting tumor growth and the inflammatory microenvironment, and serve as a promising adjunct medication.

## Introduction

Among all types of cancer, lung cancer ranks first in both incidence and mortality^1^. Early-stage lung cancer patients primarily undergo surgical treatment, whereas those in the middle and advanced stages typically receive a combination of chemotherapy, targeted therapy, immunotherapy, and radiation therapy^2^. Mannose is a natural six-carbon monosaccharide found in various fruits, plants, and fungi, and is widely present in various tissues and fluids of the human body^3^. The primary physiological function of mannose is to regulate protein glycosylation rather than to serve as an energy source^4^. Mannose also possesses potent anti-inflammatory properties, and several clinical trials have demonstrated its application as an adjuvant treatment for urinary tract infections, with a favorable safety profile^5^, ^6^. Gonzalez *et al*. were the first to report that mannose has potent antitumor effects and enhances the efficacy of chemotherapy in patients with osteosarcoma and pancreatic cancer^7^. A subsequent study demonstrated that mannose also enhances the efficacy of immunotherapy and radiotherapy via degradation of PD-L1 in triple-negative breast cancer^8^. Notably, these antitumor effects of mannose are achieved by simple oral administration of mannose via drinking water, without significantly affecting the health or weight of tumor bearing mice^9^. Taken together, these findings indicate that mannose has promising potential to serve as a novel adjuvant antitumor therapy for cancer patients. However, the antitumor effects of mannose in non-small cell lung cancer (NSCLC) have not been fully elucidated.

Chronic inflammatory responses are a hallmark of the tumor microenvironment^10^. Cancer cells can hijack inflammatory mechanisms to promote their own growth and survival, a process considered essential for tumor development. The tumor inflammatory microenvironment is characterized by the extensive infiltration of inflammatory cells such as tumor-associated macrophages and neutrophils, and the massive expression of inflammatory cytokines such as IL-6 and IL-8^11^. IL-8, a cytokine that specifically attracts and activates immunosuppressive cells such as neutrophils and myeloid-derived suppressor cells, plays a pivotal role in multiple physiological and pathological processes, including immune responses and inflammatory reactions^12^. In patients with lung cancer, serum IL-8 levels are abnormally elevated and positively correlated with the tumor burden but inversely related to the benefits obtained by immunotherapy^13^, ^14^. Whether mannose can suppress the inflammatory microenvironment in NSCLC by inhibiting IL-8 expression requires further investigation.

In recent years, the unique role of the gut microbiota in the development and progression of tumors has garnered increasing attention from researchers^15^. Significant differences in the composition and abundance of the gut microbiota are observed between cancer patients and healthy individuals, and among patients with different types of cancer^16^. Certain gut microbes and their harmful metabolic byproducts can directly promote cancer development, whereas others may indirectly contribute to cancer progression by inducing inflammation and suppressing immune functions^17^. Conversely, some gut microbes, recognized as probiotics, have been shown to enhance immunity, suppress tumor development and improve the efficacy of clinical treatments^18^. The regulatory effects of orally administered mannose on the gut microbiota in NSCLC have not been reported.

In this study, we aimed to investigate the regulatory effects of mannose in NSCLC. We demonstrated that mannose treatment can inhibit cancer cell growth and inflammatory microenvironment formation in NSCLC tissue, and enhance the antitumor efficacy of immune checkpoint inhibitor. On the one hand, orally administered mannose significantly reshaped the gut microbiota, blood and fecal metabolite profiles of NSCLC-bearing mice. On the other hand, mannose targeted OGT and inhibited the downstream hnRNP R/JUN/IL-8 axis in NSCLC cells. Our data indicate the potent antitumor efficacy of mannose, and its potential application as an adjuvant therapy for NSCLC patients.

## Materials and Methods

### Cell culture

The mouse Lewis lung carcinoma cell line LLC; the human NSCLC cell lines A549, PC-9, H1299 and SK-MES-1; and the human bronchial epithelial cell line 16HBE were purchased from the cell bank of the Chinese Academy of Sciences. LLC, A549, and SK-MES-1 cells were cultured in DMEM (Gibco, USA), and PC-9, H1299, and 16HBE cells were cultured in RPMI 1640 medium (Gibco), supplemented with 10% fetal bovine serum (Gibco) and 1% antibiotics (Gibco), at 37°C/5% CO_2_ in a humidified atmosphere. Cells were cultured within ten passages post-thaw and were passaged every 2-3 days.

### Animal studies

The animal studies were approved by the local ethics committee of First Affiliated Hospital of Guangzhou Medical University (20240076), and all the animal handling procedures were conducted in accordance with standard procedures. Subcutaneous tumor models were established by injecting 1×10^6^ LLC cells or 5×10^6^ A549 cells into the flanks of 4-6 weeks C57BL/6 female mice or 4-6 weeks BALB/c female nude mice (Huafukang, China), which were maintained in a specific-pathogen-free animal housing facility. For gut microbiota and metabolomic analyses, LLC-bearing C57BL/6 mice were treated with drinking water containing 20% mannose (Beyotime, China) or with pure drinking water from day 8 to day 21. For tumor growth and inflammatory microenvironment analysis, A549 tumor-bearing BALB/c nude mice were treated with drinking water containing 20% mannose or with pure drinking water from day 8 to day 30. For combination therapy analysis, LLC-bearing C57BL/6 mice were treated with drinking water containing 20% mannose, an anti-mouse Pd-1 antibody (100 μg per mouse, twice a week, MCE, China) or a combination of mannose and anti-mouse Pd-1 from day 8 to day 30. The size of the implanted tumors was measured via a Vernier caliper (tumor volume = (L×W^2^)/2, L: tumor long axis, W: tumor short axis).

### Western blotting assay

Protein sample preparation and western blotting were performed as previously described^19^. Antibodies against MPI (ProteinTech, USA), JUN (ProteinTech), O-GlcNAc (ProteinTech), OGT (ProteinTech), hnRNP R (ProteinTech), hnRNP U (ProteinTech), hnRNP Q (ProteinTech), hnRNP A3 (ProteinTech), hnRNP A2/B1 (ProteinTech) and β actin (ProteinTech) were used.

### Quantitative reverse transcriptase polymerase chain reaction (qRT‒PCR)

RNA sample preparation and qRT‒PCR were performed as previously described^20^. Primers for MPI, JUN, hnRNP R and hnRNP A2/B1 were used (Sangon, China). The sequences of the primers used were as follows: MPI: forward TGGGTTCCAACAGCGAAGTG, reverse CATAAGGCTTGTCCTCTGCGA; JUN: forward TGTACCGACTGAGAGTTCTTGA, reverse ACAGAGCGAGTGAAAATGTGTAT; hnRNP R: forward GCAAGGTGCAAGAGTCCACA, reverse CACGCCAGAGTACACACTGTC; hnRNP A2/B1: forward ATTGATGGGAGAGTAGTTGAGCC, reverse AATTCCGCCAACAAACAGCTT; and GAPDH: forward GGAGCGAGATCCCTCCAAAAT, reverse GGCTGTTGTCATACTTCTCATGG.

### Cell viability assay

The viability of H1299, A549, SK-MES-1, and PC-9 cells treated with different concentrations (0, 11, 25, 50, 75, or 100 mM) of mannose for different durations (12, 24, 36 and 48 hours) was analyzed with a Cell Counting Kit-8 (Dojindo, Japan) following the manufacturer's protocol.

### Colony formation assay

H1299, A549, SK-MES-1, and PC-9 cells (1000 cells per well) were seeded into 6-well plates, and different concentrations (0, 11, 25, 50, 75, 100 mM) of mannose, or culture supernatants of *Lactobacillus intestinalis* (5%) or *Lactobacillus acidophilus* (5%) were added to the culture medium. The cells were stained with crystal violet and observed after 7 days of culture.

### Apoptosis assay

H1299, A549, SK-MES-1, and PC-9 cells were treated with different concentrations (0, 11, 25, 50, 75, or 100 mM) of mannose for 48 hours. The percentage of apoptotic cells was analyzed via flow cytometry via an apoptosis assay kit (Solarbio, China) following the manufacturer's protocol.

### Immunohistochemistry

Immunohistochemistry analysis of paraffin-embedded NSCLC tissue sections was performed with antibody against Ki67 (ProteinTech) as previously described^20^.

### Immunofluorescence

Immunofluorescence analysis of paraffin-embedded NSCLC tissue sections was performed as previously described^21^. Antibodies against CD11b (ProteinTech), Ly-6G (ProteinTech), F4/80 (ProteinTech) and CD8 (ProteinTech) were incubated overnight at 4°C, followed by incubation with APC-conjugated secondary antibodies (Abcam, UK) and DAPI (Beyotime), and the results were visualized with a fluorescence microscope. The cells cultured on cover slides were fixed, blocked, incubated with antibodies against OGT (Proteintech), hnRNP R (Proteintech) at 4°C overnight, incubated with fluorescein (FITC)-conjugated secondary antibody (Proteintech) or streptavidin-FITC (Beyotime) and DAPI (Beyotime), and visualized with a fluorescence microscope.

### Flow cytometry

A cell suspension was prepared from mechanically and enzymatically dissociated tumor tissue and adjusted to a concentration of 1×10^6^ cells/mL as previously described^22^. For flow cytometry analysis, approximately 1×10^6^ cells were resuspended in 100 µL of staining buffer (PBS with 2% FBS), incubated with antibodies against CD11b (ProteinTech), Ly-6G (ProteinTech), F4/80 (ProteinTech), CD86 (Proteintech), and CD206 (Abcam) overnight at 4°C, followed by incubation with a FITC-conjugated secondary antibody (Proteintech). For IL-8 analysis, the cells were fixed with 4% paraformaldehyde and permeabilized with Triton X-100 before being incubated with an anti-IL-8 antibody (Abcam). Flow cytometry was performed via a Becton Dickinson Biosciences flow cytometry system (BD Biosciences, USA), and the data were analyzed with FlowJo software. The mean fluorescence intensity was quantified, and statistical significance was determined via Student's t test (p < 0.05 was considered statistically significant).

### Enzyme-linked immunosorbent assay (ELISA)

Serum IL-1β, IL-6, IL-8, TNF-α, and IFN-α levels in NSCLC-bearing mice were measured with a mouse IL-1β ELISA kit (Beyotime), a mouse IL-6 ELISA kit (Beyotime), a human IL-8 ELISA kit (Beyotime), a mouse TNF-α ELISA kit (Beyotime), and a mouse IFN-α ELISA kit (Cloud Clone, China) following the manufacturers' instructions. Serum samples were diluted in advance with PBS (Gibco). IL-8 levels in the supernatants of NSCLC cells were measured with a human IL-8 ELISA kit (Beyotime) following the manufacturer's instructions. The supernatants were concentrated in advance via ultrafiltration centrifuge tubes (Millipore, USA) following the manufacturer's instructions.

### Metagenomic sequencing

Metagenomic sequencing was performed by Shanghai Applied Protein Technology (China). In brief, total DNA was extracted from the intestinal contents of tumor-bearing mice via a magnetic soil and stool DNA kit (TIANGEN, China) according to the manufacturer's instructions. The sequencing libraries were generated via the NEBNext® Ultra™ DNA Library Prep Kit for Illumina (NEB, USA) following the manufacturer's instructions, and index codes were added to attribute the sequences to each sample. Clustering of the index-coded samples was performed on a cBot Cluster Generation System according to the manufacturer's instructions. After cluster generation, the library preparations were sequenced on an Illumina NovaSeq 6000 platform. Raw data of metagenomic sequencing were uploaded to the Sequence Read Archive (SRA) database (ID: PRJNA1203192).

### Metabolomic analysis

Metabolomic analysis of blood and feces samples was performed by Shanghai Applied Protein Technology. In brief, samples were separated with an Agilent 1290 Infinity LC ultrahigh-performance liquid chromatography system and subjected to mass spectrometry analysis via a Triple TOF 6600 mass spectrometer (AB SCIEX, USA), with detection conducted in both positive and negative ion modes via electrospray ionization. Sample primary and secondary spectra were collected via a Triple TOF 6600 mass spectrometer (AB SCIEX).

### Transcriptome sequencing

Transcriptome sequencing of mannose-treated H1299 cells was performed by Shanghai Applied Protein Technology. In brief, transcriptome libraries were prepared via a TruSeqTM RNA sample preparation kit (Illumina). Double-stranded cDNA was synthesized via a SuperScript double-stranded cDNA synthesis kit (Invitrogen) with random hexamer primers (Illumina). The paired-end RNA library was quantified with TBS380 and sequenced via an Illumina HiSeq X Ten/NovaSeq 6000 sequencer. Raw data of transcriptome sequencing were uploaded to the SRA database (ID: PRJNA1203014).

### Bioinformatics analysis

All of the analyses were performed via an in-house pipeline from Shanghai Applied Protein Technology. In brief, gene prediction and abundance analysis were performed with Prodigal, CD-HIT and Bowtie2 software. Taxonomic analysis was performed with DIAMOND, MEGAN and Gephi software. Functional analysis was performed with DIAMOND software and the KEGG, eggNOG and CAZy databases. Resistance gene annotation was performed with ARG-OAP v2.0 software and the SARG2 database. PCA decrease-dimension analysis was performed with the R ade4 package (version 2.15.3), and NMDS decrease-dimension analysis was performed with the R vegan package (version 2.15.3). Statistical analysis was performed with ANOSIM and Adonis analysis (R vegan package, version 2.15.3), Wilcoxon (two groups), Kruskal‒Wallis (multiple groups), the rank sum test (or STAMP analysis) and LEfSe analysis. Survival analysis was performed with the GEPIA website (http://gepia.cancer-pku.cn/index.html). Tumor infiltrating immune cells were analyzed with the TIMER website (https://cistrome.shinyapps.io/timer/).

### Coimmunoprecipitation (Co-IP)

For Co-IP analysis, samples were prepared with an O-GlcNAc antibody (ProteinTech) and an immunoprecipitation kit (Beyotime) following the manufacturer's instructions. Binding proteins were analyzed via western blotting with corresponding antibodies (hnRNP R, hnRNP U, hnRNP Q, hnRNP A3, hnRNP A2/B1) or mass spectrometry (Thermo Fisher, USA).

### RNA immunoprecipitation (RIP)

For RIP analysis, samples were prepared with an hnRNP R antibody (ProteinTech), an hnRNP A2/B1 (ProteinTech) antibody and a BeyoRIP™ RIP assay kit (Beyotime) following the manufacturer's instructions. Binding mRNAs were detected via qRT‒PCR analysis with a JUN mRNA primer (Sangon).

### Fluorescence *in situ* hybridization (FISH)

FISH analysis of cells cultured on cover slides was performed via a fluorescence *in situ* hybridization kit for RNA (Beyotime), followed by incubation with a JUN mRNA probe (Sangon), incubation with DAPI (Beyotime), and visualization with a fluorescence microscope.

### RNA pull-down

For RNA pull-down analysis, samples were prepared with a JUN mRNA sense (Sangon) and RNA protein pull-down kit (Sangon) following the manufacturer's instructions. The binding proteins were detected via western blotting with an hnRNP R antibody (Proteintech).

### mRNA stability assay

NSCLC cells subjected to different experimental treatments (+25 mM mannose, hnRNP R knockdown, +25 mM mannose and O-linked N-acetylglucosamine transferase (OGT) overexpression, normal control) were supplemented with actinomycin D, and the expression level of JUN mRNA was analyzed via qRT‒PCR at 0, 6, 12, 18, and 24 h.

### Small molecule pull-down

Small molecule pull down was performed as previously described^22^. In brief, lysates of A549 and H1299 cells were incubated with biotin tagged mannose before being captured with streptavidin magnetic beads (Beyotime). The captured samples were further analyzed with western blotting with antibody against OGT.

### Cellular thermal shift assay (CETSA)

CETSA assay was performed as previously described^20^. In brief, A549 and H1299 cells were treated with 20 mM mannose for 12 h before being harvested. The harvested samples were further dispensed into PCR tubes (100 µl per tube) and heated at 37, 41, 45, or 49°C for 3 min. The heated samples were cooled to 4°C and analyzed via western blotting with an antibody against OGT.

### Molecular docking and molecular dynamics simulation

Molecular docking analysis was performed with AutoDock software (version 4.2.6). Molecular dynamics simulation, including root mean square deviation (RMSD), root mean square fluctuation (RMSF), radius of gyration, and solvent-accessible surface area (SASA), was performed with GROMACS software (version 2024.03).

### Lung cancer organoid culture and Calcein/PI cell viability and cytotoxicity assay

The study was approved by the local ethics committee of Liaoning Cancer Hospital& Institute (GZR20240373), and written informed consent was obtained from all subjects. Lung cancer tissues were obtained during surgical procedures and were minced into small pieces and further digested with Cebrary ® tissue digestion solution (Yeasen, China). Digested cell suspension was filtered and resuspended with Matrigel (1:1, Corning, USA) before being seeded into culture plates. Organoid was cultured with Cebrary® lung cancer organoid growth medium (Yeasen), and the culture medium was refreshed every two days. Calcein/PI cell viability and cytotoxicity assay was performed with a Calcein/PI cell viability/cytotoxicity assay kit (Beyotime) following the manufacturer's protocol.

### Statistical analysis

Statistical analysis was performed, and charts were generated via GraphPad Prism 7 software. All the data are presented as the means ± SDs of at least three independent experiments. Differences between the indicated groups were analyzed with Student's t test, and p<0.05 was considered statistically significant (p<0.05 marked *, p<0.01 marked **, p<0.001 marked ***, p<0.0001 marked ****).

## Results

### Mannose inhibits NSCLC cell growth *in vitro* and *in vivo*

**Figure 1:**
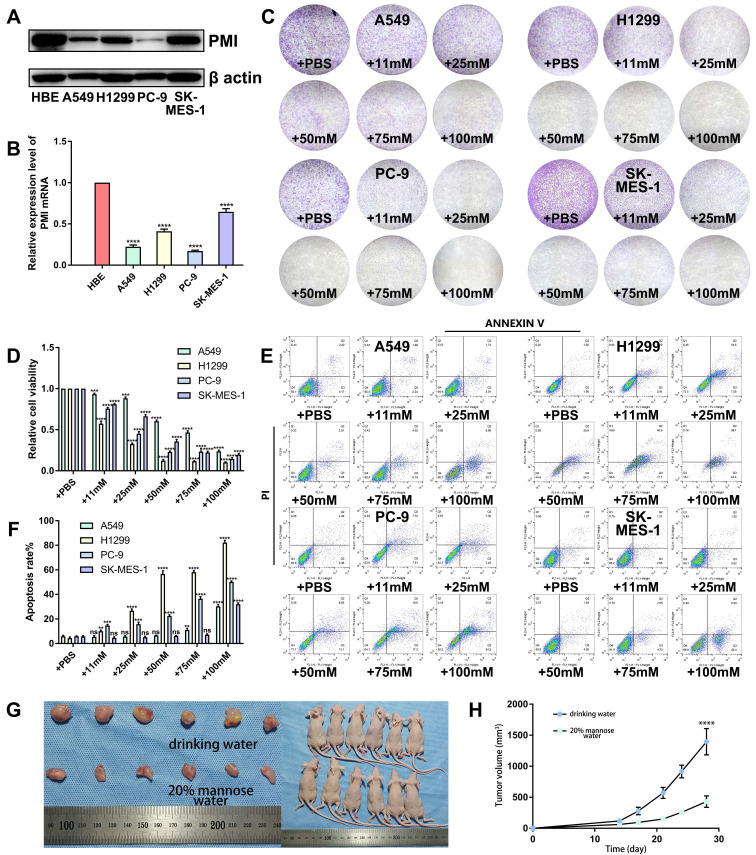
Mannose inhibits NSCLC cell growth *in vitro* and *in vivo*. (A-B) Western blotting and qRT‒PCR analysis of MPI protein and mRNA expression in human NSCLC cell lines (H1299, A549, SK-MES-1, and PC-9) and human bronchial epithelial (HBE) cells. (C-D) Colony formation analysis of H1299, A549, SK-MES-1, and PC-9 cells treated with different concentrations of mannose. (E-F) Apoptosis analysis of H1299, A549, SK-MES-1, and PC-9 cells treated with different concentrations of mannose. (G-H) Mannose inhibited the growth of transplanted A549 cells in nude mice (n=6).

To verify the antitumor efficacy of mannose in NSCLC, we first assessed the protein and mRNA expression levels of MPI in human NSCLC cell lines (H1299, A549, SK-MES-1, and PC-9) and human bronchial epithelial (HBE) cells via western blotting and qRT‒PCR. The results revealed that MPI expression was generally lower in NSCLC cells than in HBE cells (Figure 1A-1B). Next, we cultured the aforementioned NSCLC cells with different concentrations (0, 11, 25, 50, 75, and 100 mM) of mannose and determined IC50 (half maximal inhibitory concentration) values (Supplementary Figure 1). Colony formation assay revealed that 11 to 25 mM mannose significantly inhibited the growth of NSCLC cells (Figure 1C-1D). Additionally, apoptosis assay revealed that 11 to 25 mM mannose significantly induced the apoptosis of NSCLC cells (Figure 1E-1F). However, there was no absolute negative correlation between MPI expression levels and the antitumor effects of mannose. We further established a subcutaneous tumor model in nude mice via A549 cells, and the mice were treated with drinking water containing 20% mannose from days 8 to 30. The results demonstrated that mannose effectively inhibited the growth of the transplanted tumors (Figure 1G-1H). Taken together, these results indicate that mannose can inhibit NSCLC cell growth both *in vitro* and *in vivo*.

### Mannose suppresses the formation of an inflammatory microenvironment in an NSCLC mouse model

**Figure 2:**
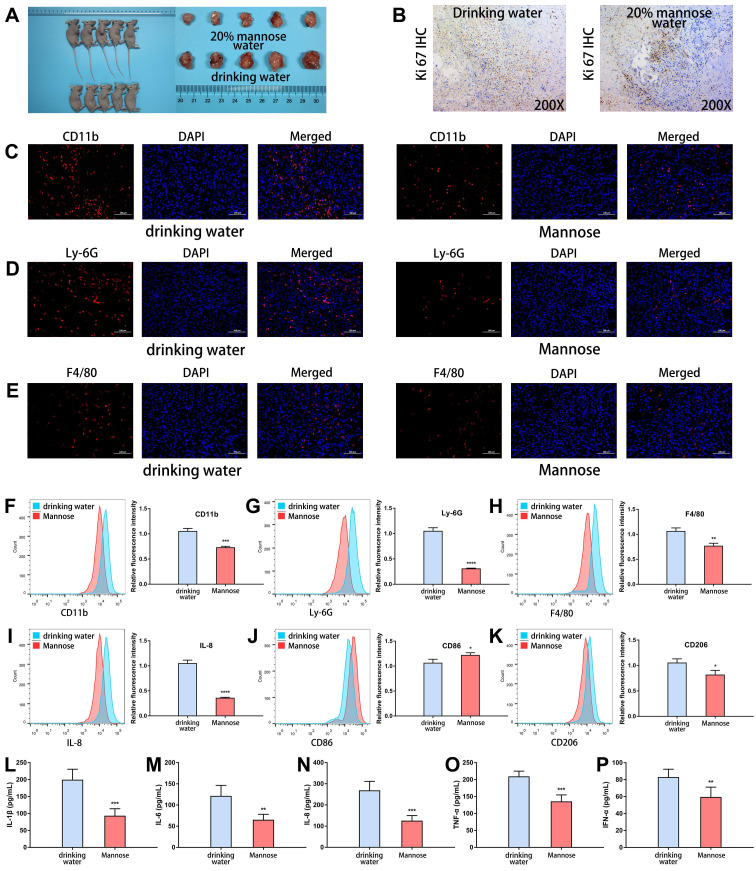
Mannose suppresses the formation of an inflammatory microenvironment in an NSCLC mouse model. (A) Mannose inhibits the growth of transplanted A549 cells in nude mice (n=5). (B) Mannose decreases Ki-67 expression in A549 cells transplanted into nude mice. (C-E) Immunofluorescence analysis of myeloid cell, neutrophil and macrophage infiltration in transplanted tumors via analysis of CD11b, Ly-6G and F4/80 expression. (F-H) Flow cytometry analysis of myeloid cell, neutrophil and macrophage infiltration in transplanted tumors via analysis of CD11b, Ly-6G and F4/80 expression. (I) Flow cytometry analysis of IL-8 expression in transplanted tumors. (J) Flow cytometry analysis of M1 macrophage polarization in transplanted tumors via analysis of CD86 expression. (K) Flow cytometry analysis of M2 macrophage polarization in transplanted tumors via analysis of CD206 expression. (L‒P) ELISA analysis of the serum IL-1β, IL-6, IL-8, TNF-α, and IFN-α levels in A549-transplanted nude mice treated with mannose or drinking water.

Next, we explored the regulatory roles of mannose in the inflammatory microenvironment of transplanted tumor tissues. As expected, mannose treatment significantly inhibited the growth of the transplanted tumors, as assessed by tumor size measurement and Ki-67 staining (Figure 2A-2B). Immunofluorescence analysis of the resected transplanted tumors revealed that mannose treatment reduced CD11b expression within the tumor, indicating decreased myeloid cell infiltration (Figure 2C). More specifically, mannose treatment reduced Ly6G and F4/80 expression in tumors, indicating decreased neutrophil and macrophage infiltration (Figure 2D-2E). To further verify these results, we performed flow cytometry analysis on the resected transplanted tumors. The results demonstrated that mannose reduced CD11b, Ly6G, F4/80, and IL-8 expression, indicating decreased neutrophil and macrophage infiltration within the tumor (Figure 2F-2I). Additionally, mannose increased M1 macrophage polarization and decreased M2 macrophage polarization (Figure 2J-2K). Mannose treatment also significantly decreased the serum expression levels of multiple inflammatory cytokines, such as IL-1β, IL-6, IL-8, TNF-α and IFN-α (Figure 2L-2P). In summary, these findings demonstrate that, in addition to its direct antitumor effects, mannose can suppress the formation of an inflammatory microenvironment in NSCLC.

### Mannose reshaped the gut microbiota in an NSCLC mouse model

**Figure 3:**
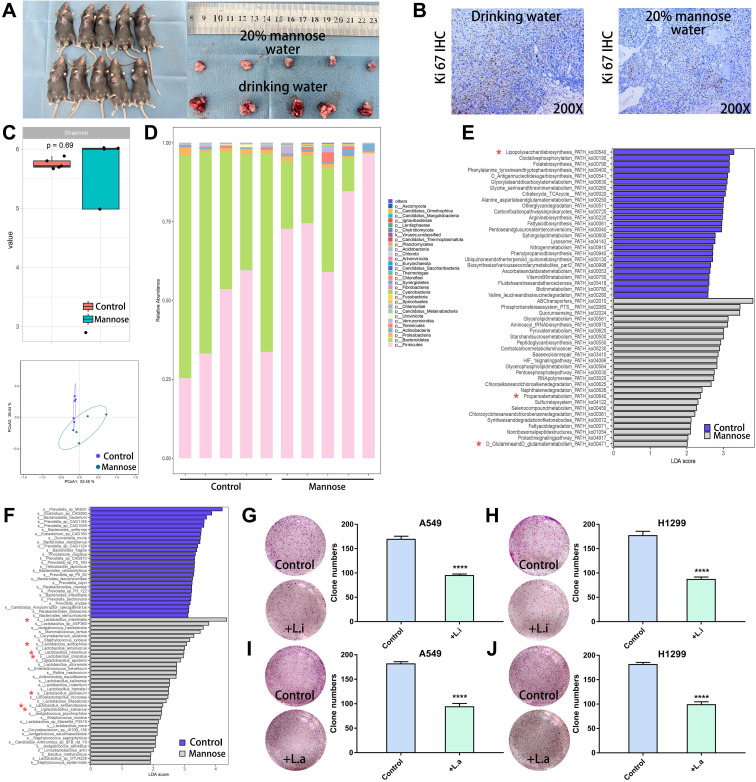
Mannose reshaped the gut microbiota in an NSCLC mouse model. (A) Mannose inhibits the growth of transplanted LLC cells in C57BL/6 mice (n=5). (B) Mannose decreased Ki-67 expression in transplanted LLC cells from C57BL/6 mice. (C) Shannon index and principal coordinate analysis of the gut microbiota in control and mannose-treated LLC-transplanted C57BL/6 mice. (D) Heatmap of the identified gut microbiota at the phylum level in control and mannose-treated LLC-transplanted C57BL/6 mice. (E) Functional enrichment pathway analysis of the identified differential microbiota by LEfSe analysis. (F) LEfSe analysis of the identified differential microbiota at the species level in control and mannose-treated LLC-transplanted C57BL/6 mice. (G-J) Colony formation analysis of A549 and H1299 cells supplemented with 5% culture supernatants of *Lactobacillus intestinalis* (*L.i.*) or *Lactobacillus acidophilus* (*L.a.*). Identified key pathways and microbiota species were labeled with *.

To explore the regulatory effects of mannose on the gut microbiota and metabolites in NSCLC, we established a subcutaneous tumor model in C57BL/6 mice with LLC cells. The tumor-bearing mice were treated with daily drinking water containing 20% mannose or with pure drinking water from day 8 to day 21. Mannose treatment significantly inhibited the growth of the transplanted tumors, as assessed by tumor size measurement and Ki-67 staining (Figure 3A-3B). To investigate whether mannose modulates the gut microbiota in NSCLC, we performed shotgun metagenomic sequencing analysis of the intestinal contents of tumor-bearing mice (Supplementary Table 1). Principal coordinate analysis revealed that mannose treatment significantly reshaped the gut microbiota, and a species abundance clustering heatmap revealed a noticeable difference at the phylum level between the mannose and control groups (Figure 3C-3D). Functional enrichment pathway analysis of the identified differential microbiota revealed that the mannose treatment group was enriched in propionate and glutamine metabolic pathways, which are known for their immune-boosting and antitumor properties^23^, ^24^. In contrast, the control group was enriched in metabolic pathways related to lipopolysaccharides (Figure 3E), substances closely associated with inflammation and bacterial infection^25^. These results suggest that mannose can modulate the gut microbiota to enhance biological functions such as immunity and antitumor, anti-inflammatory, and antibacterial activities. Further analysis at the species level revealed that the addition of mannose significantly increased the proportion of probiotics, such as *Lactobacillus intestinalis*, *Lactobacillus acidophilus*, and *Ligilactobacillus salivarius,* in the microbiota (Figure 3F). To further validate the biological functions of the identified probiotics, we supplemented the culture supernatants of *Lactobacillus intestinalis* (5%) and *Lactobacillus acidophilus* (5%), which significantly inhibited the growth of the human NSCLC cell lines A549 and H1299 (Figure 3G-3J). These findings demonstrate that mannose effectively reshapes the gut microbiota and increases the proportion of probiotics, which in turn contributes to enhanced immunity and antitumor, anti-inflammatory, and antibacterial effects.

### Mannose alters blood and fecal metabolites in an NSCLC mouse model

**Figure 4:**
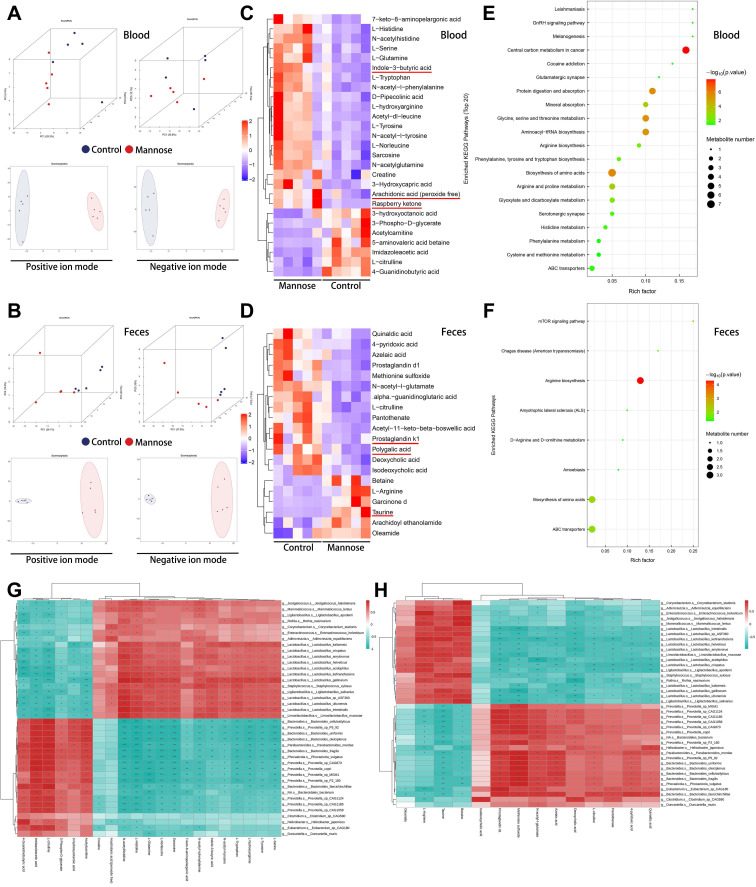
Mannose alters blood and fecal metabolites in an NSCLC mouse model. (A-B) Principal coordinate analysis of the metabolic profiles of blood and feces from control and mannose-treated LLC-transplanted C57BL/6 mice. (C-D) Heatmap of the identified differential blood and fecal metabolites in control and mannose-treated LLC-transplanted C57BL/6 mice. (E-F) Functional enrichment pathway analysis of the identified differential blood and fecal metabolites via a ballon plot. (G) Heatmap of the correlations between the identified differential microbiota and blood metabolites according to Spearman analysis. (H) Heatmap of the correlations between the identified differential microbiota and fecal metabolites according to Spearman analysis. Identified key metabolites were underlined with red line.

Given the crucial role of the gut microbiota in digestion and metabolism and the significant influence of microbial metabolites on physiological and pathological activities, we conducted metabolomic analyses of blood and feces from tumor-bearing mice after mannose treatment (Supplementary Tables 2-5). Principal component analysis and partial least-square discriminant supervised analysis revealed significant separation in the metabolic profiles of the blood and feces between the mannose treatment group and the control group, indicating that mannose treatment effectively remodeled the metabolite profile in these samples (Figure 4A-4B). Mannose treatment notably increased the abundance of metabolites with immune-boosting and antitumor properties, such as butyric acid, arachidonic acid, and raspberry ketone in the blood, as well as taurine in the feces. Additionally, it significantly reduced the levels of proinflammatory metabolites such as prostaglandins in the feces (Figure 4C-4D). Functional enrichment pathway analysis revealed that the differentially abundant metabolites were enriched in pathways related to central carbon metabolism, amino acid biosynthesis, and arginine biosynthesis, suggesting that mannose regulates the gut microbiota to modulate the metabolism of sugars and amino acids in mice (Figure 4E-4F). To further investigate whether the changes in metabolite abundance in blood and feces are potentially related to alterations in the gut microbiota, we conducted a combined bioinformatics analysis of the identified differential gut microbiota and differentially abundant metabolites. The results revealed a significant positive correlation between the abundance and functional enrichment pathways of the identified differential blood and fecal metabolites and gut microbes (Figure 4G-4H, Supplementary Figure 2A-2C). These results indicated that changes in the abundance of the gut microbiota led to alterations in metabolic activity, which in turn played a pivotal role in the changes in metabolite abundance in the blood and feces. In summary, our results indicate that mannose increases the proportion of beneficial bacteria in the gut microbiota, thereby increasing the abundance of immune-boosting and antitumor metabolites in the blood and feces while reducing the abundance of proinflammatory metabolites, ultimately promoting immune function and antitumor and anti-inflammatory effects.

### Mannose inhibits the JUN/IL-8 signaling axis in NSCLC cells

**Figure 5:**
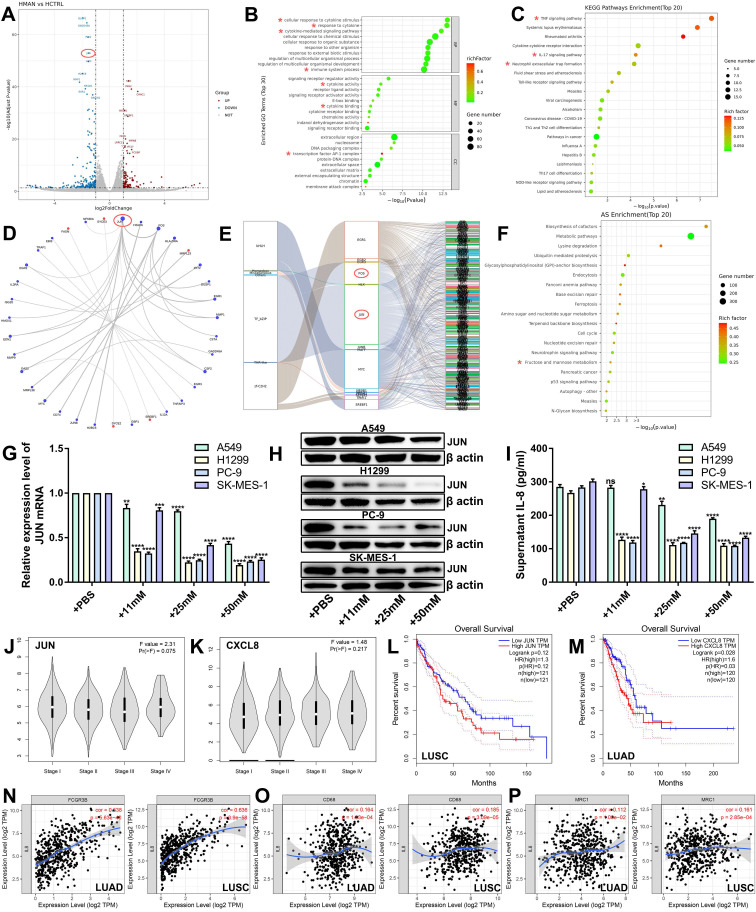
Mannose inhibits the JUN/IL-8 signaling axis in NSCLC cells. (A) Volcano plot of the identified differentially expressed genes (DEGs) in H1299 cells treated with mannose or PBS. JUN was significantly downregulated in mannose-treated H1299 cells. (B) Gene Ontology analysis of the identified DEGs via a ballon plot. (C) Functional enrichment pathway analysis of the identified DEGs via a ballon plot. (D) Interaction network analysis of the top 30 identified DEGs, indicating that JUN is a central node. (E) Transcription factor analysis of the identified DEGs, indicating that JUN and FOS are core transcription factors. (F) Alternative splicing enrichment analysis of the identified DEGs, indicating significant enrichment in the fructose and mannose metabolism pathways. (G) qRT‒PCR analysis of IL-8 mRNA expression in H1299, A549, SK-MES-1, and PC-9 cells treated with different concentrations of mannose. (H) Western blot analysis of IL-8 protein expression in H1299, A549, SK-MES-1, and PC-9 cells treated with different concentrations of mannose. (I) ELISA analysis of IL-8 secretion in H1299, A549, SK-MES-1, and PC-9 cells treated with different concentrations of mannose. (J-K) Expression levels of JUN and CXCL8 in different stages of lung cancer according to the TCGA database. (L-M) Overall survival analysis of lung cancer patients with different JUN and CXCL8 expression levels in the TCGA database. (N-P) Correlations between the expression levels of CXCL8 and FCGR3B, CD68, CD206 in the TIMER database. Identified key pathways and biological functions were labeled with *.

To investigate the molecular mechanisms of the antitumor effects of mannose, we conducted transcriptome sequencing analysis on H1299 cells treated with mannose (Supplementary Table 6). The results indicated a significant downregulation of JUN expression, along with other oncogenes, such as MMP1 and EGR1 (Figure 5A). Bioinformatics analysis of the differentially expressed genes (DEGs) revealed significant enrichment in biological functions and signaling pathways related to immune-related signaling pathways, cytokine activity, cytokine-mediated signaling pathways and the AP-1 transcription factor complex (Figure 5B-5C). Interaction network analysis of the top 30 DEGs suggested that JUN is a central node (Figure 5D). Transcription factor analysis revealed JUN and FOS as core transcription factors among the DEGs (Figure 5E). Alternative splicing enrichment analysis revealed significant enrichment in the fructose and mannose metabolism pathways (Figure 5F). Using qRT‒PCR, western blotting, and ELISA, we measured the mRNA and protein levels of JUN, as well as IL-8 secretion, in NSCLC cells treated with various concentrations of mannose. The results demonstrated that mannose inhibited the mRNA and protein expression of JUN and reduced IL-8 secretion in NSCLC cells (Figure 5G-5I). These findings indicate that the JUN/IL-8 signaling axis is a critical target of mannose in NSCLC cells. Bioinformatic analysis via the TCGA database revealed that the expression levels of JUN and CXCL8 are higher in stage IV lung cancer, suggesting a potential positive correlation between their expression and disease progression (Figure 5J-5K). Survival analysis indicated that higher expression levels of JUN and CXCL8 were negatively correlated with prognosis in lung cancer patients, with the negative correlation being particularly significant between JUN and lung squamous cell carcinoma patients, and between CXCL8 and lung adenocarcinoma patients (Figure 5L-5M). Further expression correlation analysis revealed a significant positive correlation between the expression levels of IL-8 and FCGR3B (a marker of neutrophils), CD68 (a marker of macrophages), CD206 (a marker of M2 macrophages) (Figure 5N-5P). These findings indicate that JUN/IL-8 signaling is significantly correlated with disease progression and the inflammatory microenvironment of NSCLC, and is a potential target of mannose in NSCLC cells.

### Mannose inhibits O-GlcNAc glycosylation of hnRNP R to decrease JUN mRNA stability in NSCLC cells

**Figure 6:**
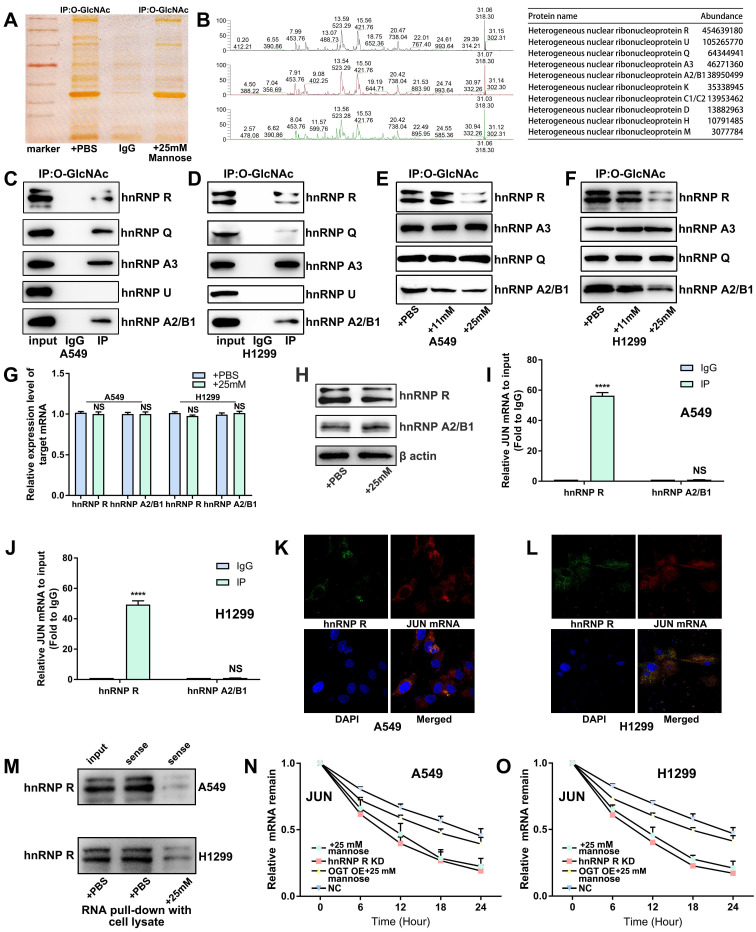
Mannose inhibits O-GlcNAc glycosylation of hnRNP R to decrease JUN mRNA stability in NSCLC cells. (A) Mannose decreased overall O-GlcNAc glycosylation in H1299 cells, as indicated by coimmunoprecipitation (co-IP) using an O-GlcNAc glycosylation antibody and subsequent silver staining. (B) MS analysis of proteins coprecipitated with the O-GlcNAc glycosylation antibody, revealing significant enrichment in the hnRNP family. (C-D) Co-IP analysis of the top 5 identified hnRNPs, indicating GlcNAc glycosylation of hnRNP R, hnRNP A3, hnRNP Q and hnRNP A2/B1. (E-F) Mannose treatment decreased the O-GlcNAc glycosylation of hnRNP R and hnRNP A2/B1 in A549 and H1299 cells. (G) Mannose treatment did not affect the overall mRNA expression of hnRNP R or hnRNP A2/B1. (H) Mannose treatment did not affect the overall protein expression of hnRNP R or hnRNP A2/B1. (I-J) RNA immunoprecipitation (RIP) analysis of hnRNP R, hnRNP A2/B1 and JUN mRNAs in A549 and H1299 cells, indicating the binding of JUN mRNA to hnRNP R but not to hnRNP A2/B1. (K-L) Immunofluorescence analysis of hnRNP R and fluorescence *in situ* hybridization analysis of JUN mRNA, indicating the colocalization of hnRNP R and JUN mRNA in A549 and H1299 cells. (M) RNA pull-down analysis of hnRNP R and JUN mRNAs, indicating that mannose treatment inhibited the binding of JUN mRNA and hnRNP R in A549 and H1299 cells. (N-O) Actinomycin D mRNA stability assay of mannose treated, hnRNP R knockdown, mannose treated and OGT overexpression, and normal control, indicating a positive association between the O-GlcNAc glycosylation of hnRNP R and the stability of JUN mRNA in A549 and H1299 cells.

Tumor cells often exhibit extensive abnormal glycosylation, which affects proteins in the cytoplasm, nucleus, and mitochondria. O-GlcNAc glycosylation, a modification involved in intracellular signaling, is notably elevated in cancer cells^26^. This abnormal glycosylation can activate oncogenic proteins, promote tumor cell proliferation and invasion, and present potential targets for anticancer therapies^27^, ^28^. Supraphysiologic concentrations of mannose have been shown to reduce O-GlcNAc glycosylation levels in tumor cells^7^. To elucidate the molecular mechanism by which mannose inhibits JUN mRNA expression in NSCLC cells, we treated H1299 cells with 25 mM mannose and performed co-IP using an O-GlcNAc glycosylation antibody followed by silver staining. The results showed that mannose treatment significantly inhibited overall O-GlcNAc glycosylation in H1299 cells (Figure 6A). We utilized mass spectrometry to identify proteins that coprecipitated with the O-GlcNAc glycosylation antibody, revealing enrichment in the hnRNP family (Figure 6B, Supplementary Table 7). We validated the five most abundant hnRNPs identified by mass spectrometry via Co-IP analysis. With the exception of hnRNP U, the remaining hnRNPs exhibited O-GlcNAc glycosylation in A549 and H1299 cells (Figure 6C-6D). Subsequent treatment of A549 and H1299 cells with various concentrations of mannose followed by co-IP analysis revealed that mannose treatment reduced O-GlcNAc glycosylation of hnRNP R and hnRNP A2/B1, but had no effect on hnRNP A3 or hnRNP Q (Figure 6E-6F). qRT‒PCR and western blotting revealed no significant changes in the mRNA or protein expression of hnRNP R or hnRNP A2/B1 after mannose treatment, suggesting that mannose mainly inhibited O-GlcNAc glycosylation rather than expression (Figure 6G-6H). Further RIP analysis demonstrated the binding of hnRNP R and JUN mRNAs, whereas hnRNP A2/B1 did not bind JUN mRNAs (Figure 6I-6J). Bioinformatics analysis of data from the TCGA database revealed no significant differences in the expression level of hnRNP R between lung cancer tissues and adjacent nontumor tissues or among different stages of lung cancer (Supplementary Figure 3A-3B). Additionally, there was no significant correlation between the expression levels of hnRNP R and overall survival or disease-free survival in patients with lung cancer (Supplementary Figure 3C-3D).

In lung cancer tissues, a weak positive correlation was observed between the expression of hnRNP R and JUN, which was not found in adjacent nontumor tissues (Supplementary Figure 3E-3F). These findings further suggest that alterations in the modification levels of hnRNP R may influence JUN expression and the progression of lung cancer. Fluorescence *in situ* hybridization and immunofluorescence experiments revealed the colocalization of hnRNP R and JUN mRNA in the cytoplasm of H1299 cells (Figure 6K-6L). RNA pull-down analysis further demonstrated that mannose treatment inhibited the binding of hnRNP R and JUN mRNAs (Figure 6M). Finally, an mRNA stability assay using actinomycin D indicated that mannose treatment decreased the mRNA stability of JUN by inhibiting hnRNP R O-GlcNAc glycosylation (Figure 6N-6O). Taken together, our results demonstrated that mannose can inhibit O-GlcNAc glycosylation of hnRNP R to decrease JUN mRNA stability in NSCLC cells.

### Mannose inhibits O-GlcNAc glycosylation in NSCLC cells by directly binding OGT

**Figure 7:**
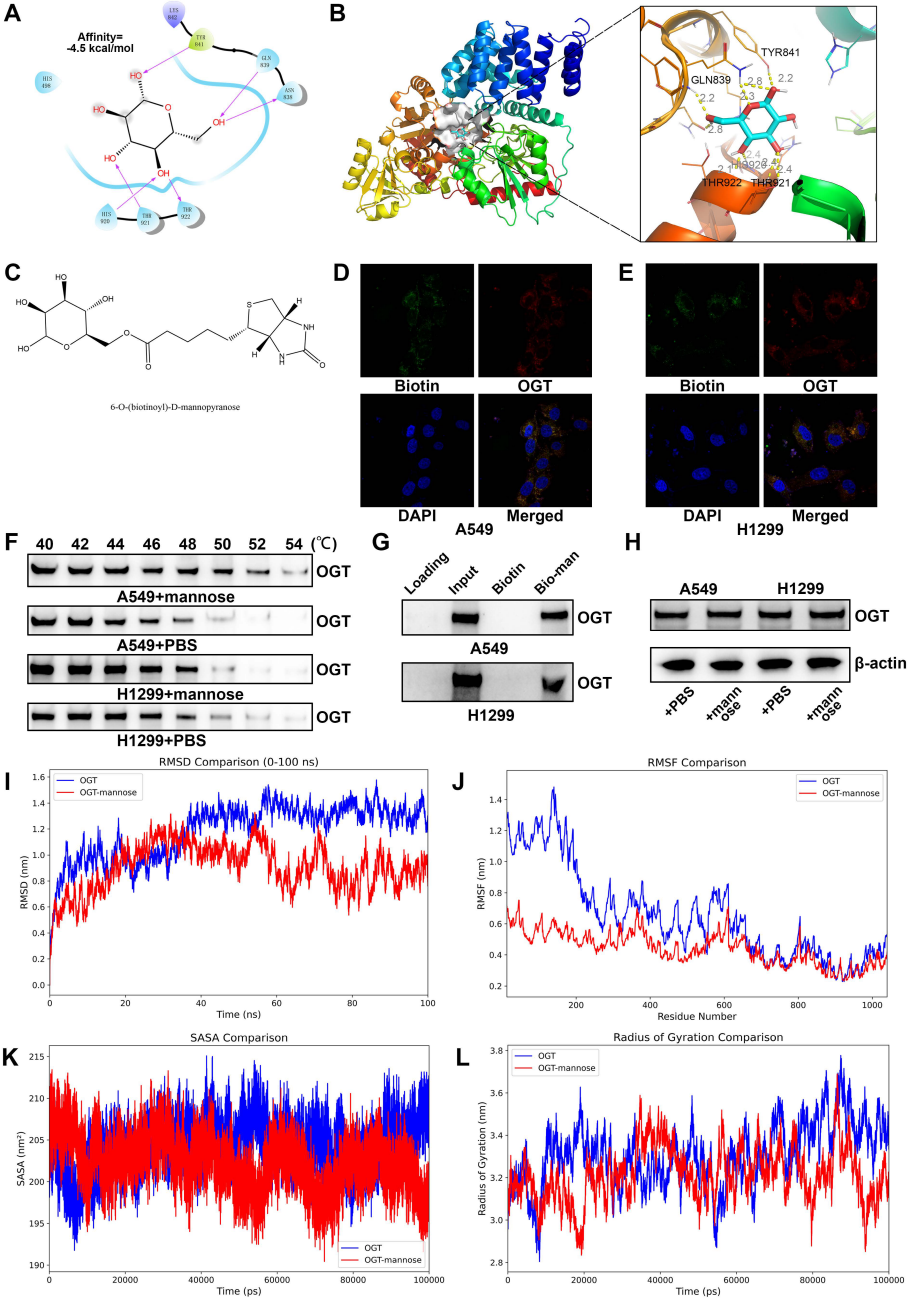
(A-B) Molecular docking analysis of the binding mode between mannose and OGT. (C) The chemical formula of biotin tagged mannose. (D-E) Immunofluorescence analysis of biotin tagged mannose and OGT, indicating the colocalization of biotin tagged mannose and OGT in A549 and H1299 cells. (F) Cellular thermal shift assay of A549 and H1299 cells treated with mannose or PBS. (G) Small molecule pull-down assay of A549 and H1299 cells treated with biotin or biotin tagged mannose. (H) Western blotting assay of A549 and H1299 cells treated with mannose or PBS. (I-L) Molecular dynamics simulations of OGT in complex with mannose.

Two main enzymes are involved in the regulation of protein O-GlcNAc glycosylation in cells: OGT and O-GlcNAcase (OGA). OGT acts as the "writer," transferring GlcNAc from the sugar donor UDP-GlcNAc to the hydroxyl oxygen atoms on serine or threonine residues of proteins, whereas OGA functions as the "eraser," hydrolyzing GlcNAc from the protein. To investigate whether mannose can inhibit O-GlcNAc glycosylation in NSCLC cells by directly binding OGT, we performed molecular docking analysis, which revealed that mannose can indeed bind OGT (Figure 7A-7B). We then tagged mannose with biotin for IF detection, and the results revealed that mannose and OGT colocalize in the cytoplasm of A549 and H1299 cells (Figure 7C-7E). Using CETSA assay, we confirmed that mannose directly binds OGT in these cells (Figure 7F). Small molecule pull-down assay further demonstrated that mannose can directly bind OGT in A549 and H1299 cells (Figure 7G). Western blotting assay demonstrated that mannose did not influence OGT protein expression (Figure 7H). Molecular dynamics simulations revealed that mannose can bind and influence the OGT conformation (Figure 7I-7L).

### Mannose inhibits NSCLC growth via the OGT/hnRNP R/JUN/IL-8 axis and synergistically enhances the antitumor efficacy of immune checkpoint inhibitors

**Figure 8:**
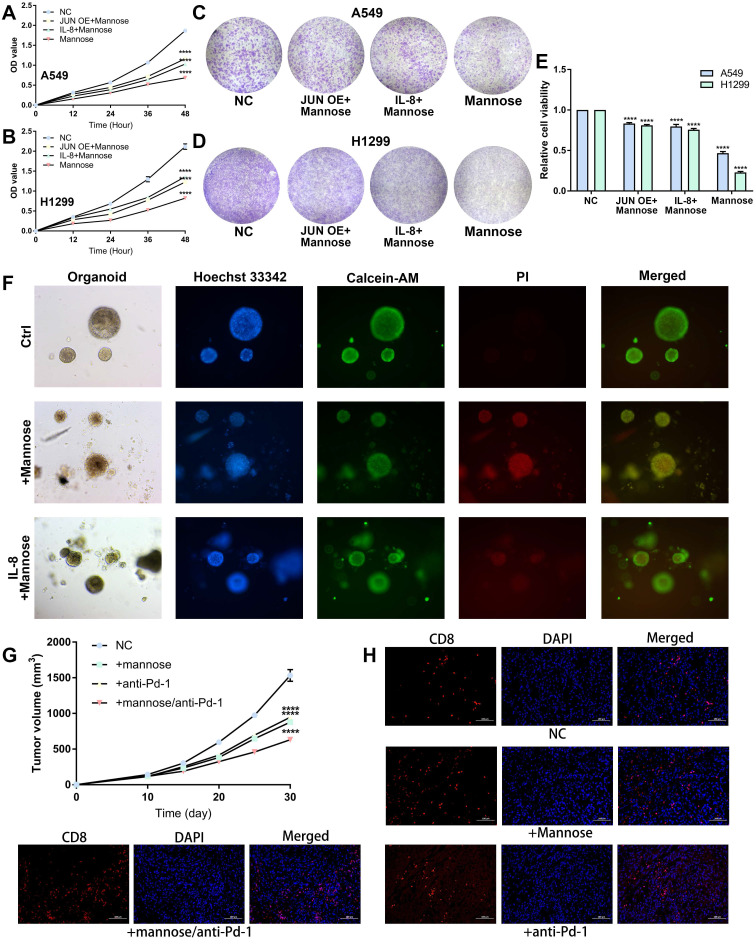
(A-B) CCK8 analysis of A549 and H1299 cells treated with mannose, mannose plus IL-8, or mannose combined with JUN overexpression. (C-E) Colony formation analysis of A549 and H1299 cells treated with mannose, mannose plus IL-8, or mannose combined with JUN overexpression. (F) Calcein/PI cell viability and cytotoxicity analysis of NSCLC organoids treated with mannose or mannose plus IL-8. (G) LLC tumor-bearing C57BL/6 mice treated with either mannose alone, a Pd-1 antibody alone, or a combination of both (n=5). (H) Immunofluorescence analysis of CD8+ T cell infiltration in transplanted tumors via analysis of CD8 expression.

On the basis of the above findings, we further confirmed that mannose inhibits NSCLC cell growth through the OGT/hnRNP R/JUN/IL-8 axis. A549 and H1299 cells were treated with mannose, mannose plus IL-8, or mannose combined with JUN overexpression, followed by CCK8 assay and colony formation assay. The results showed that both exogenous supplementation with IL-8 and JUN overexpression attenuated the inhibitory effect of mannose on NSCLC cell growth (Figure 8A-8E). Next, lung cancer organoids were treated with mannose or mannose plus IL-8, and subjected to Calcein/PI cell viability and cytotoxicity assay. The results indicated that exogenous IL-8 supplementation mitigated the inhibitory and cytotoxic effects of mannose on tumor organoids (Figure 8F). Given the significant negative correlation between the inflammatory tumor microenvironment, IL-8 expression, and the efficacy of immune checkpoint inhibitors, we further investigated whether combining mannose with immune checkpoint inhibitors could enhance antitumor effects. LLC tumor-bearing C57BL/6 mice were treated with mannose alone, a Pd-1 antibody alone, or a combination of both. The combination treatment group presented the most significant inhibition of tumor growth (Figure 8G). IF analysis of tumor tissue sections revealed increased infiltration of CD8+ T cells in both the mannose-only and the mannose plus PD-L1 inhibitor treatment groups, with the increase being more pronounced in the combination group (Figure 8H). These findings suggest that mannose can inhibit immune evasion in NSCLC cells and synergize with immune checkpoint inhibitors to enhance T cell-mediated tumor killing.

## Discussion

In this study, we first investigated the inhibitory effects of mannose on the growth of NSCLC cells. Both *in vitro* and *in vivo* functional experiments revealed that supraphysiological concentrations of mannose significantly inhibited the growth of NSCLC cells and transplanted tumors. We also demonstrated that mannose effectively reduced the infiltration of inflammatory cells such as neutrophils and macrophages, and the expression of inflammatory cytokines in tumor tissues. Considering that mannose is orally administered, we explored the regulatory effects of mannose on the gut microbiota of NSCLC-bearing mice and the results indicated that mannose significantly reshaped the gut microbiota. At the species level, the addition of mannose substantially increased the abundance of probiotics such as *Lactobacillus intestinalis*, *Lactobacillus acidophilus*, and *Ligilactobacillus salivarius*^29^, ^30^.

Functional enrichment analysis of the identified differential microbiota revealed that the propionate and glutamine metabolic pathways were enriched in the mannose-treated group, whereas the control group was enriched in the lipopolysaccharide synthesis pathway. These results suggest that mannose not only directly regulates the composition of the gut microbiota but also modulates the expression of gut microbiota-related metabolites. To validate this hypothesis, we further conducted metabolomic analyses on the blood and feces of NSCLC tumor-bearing mice. The results revealed that mannose significantly reshaped the metabolic profiles of the blood and feces of these mice. Consistent with the results of metagenomic sequencing, the levels of antitumor and anti-inflammatory metabolites such as butyrate, arachidonic acid, and raspberry ketone in the blood and taurine in the feces were significantly greater in the mannose-treated group than in the control group^31^-^34^. Conversely, the levels of proinflammatory metabolites such as prostaglandins in the feces were significantly reduced. We further conducted a combined analysis of the microbiota and metabolism and the results revealed a significant positive correlation. These results indicate the potent anti-inflammatory and antitumor effects of mannose through the reshaping of the gut microbiota and metabolite profiles, which partially explains the suppression of the inflammatory microenvironment by mannose.

Next, we applied transcriptome sequencing to further investigate the molecular mechanism by which mannose inhibits the growth and inflammatory microenvironment of NSCLC. The results revealed that JUN mRNA was one of the most significantly downregulated transcripts. JUN is the main transcription factor of the inflammatory cytokine IL-8^20^. Therefore, we investigated how mannose inhibited JUN mRNA expression. Tumor cells exhibit extensive abnormal glycosylation, such as O-GlcNAc glycosylation, which is abnormally elevated in the cytoplasm, nucleus, and mitochondria of tumor cells^27^. Abnormal O-GlcNAc glycosylation is involved in intracellular signal transduction, oncogenic protein activation and expression, tumor cell proliferation and invasion, and novel targets for antitumor drugs^26^. A supraphysiological concentration of mannose reportedly reduces O-GlcNAc glycosylation in tumor cells^8^. Thus, we explored whether mannose reduces JUN mRNA expression in NSCLC cells by regulating O-GlcNAc glycosylation. We first demonstrated that mannose significantly decreased overall O-GlcNAc glycosylation in NSCLC cells. We further identified enrichment of heterogeneous nuclear ribonucleoproteins (hnRNPs) among the binding proteins that coprecipitated with the O-GlcNAc glycosylation antibody. hnRNPs are a highly conserved family of RNA-binding proteins that participate in cellular transcription, posttranscriptional modifications, and translation through mechanisms such as regulating mRNA stability, alternative splicing, and acting as co-transcription factors^35^. We demonstrated that hnRNP R can bind and stabilize JUN mRNA in an O-GlcNAc glycosylation dependent manner, and that mannose can decrease hnRNP R O-GlcNAc glycosylation and, subsequently, JUN mRNA stability by targeting OGT. Subsequent functional experiments with NSCLC cell lines and organoids validated that mannose can inhibit cancer cell growth by targeting the OGT/hnRNP R/JUN/IL-8 axis. Finally, we observed that the combination of mannose and immune checkpoint inhibitor enhanced T-cell immune activity, thereby increasing the antitumor efficacy of immune checkpoint inhibitors. Current progress on the clinical application of mannose in cancer was summarized in our previous review^9^.

## Conclusions

This study is the first to report that mannose can inhibit NSCLC growth and the inflammatory microenvironment by reshaping the gut microbiota and inhibiting the OGT/hnRNP R/JUN/IL-8 axis. Our study indicates that mannose can serve as a promising adjunct medication for NSCLC patients.

## Supplementary Material

Supplementary figures and tables.

## Abbreviations

CETSA: cellular thermal shift assay
Co-IP: coimmunoprecipitation
ELISA: enzyme-linked immunosorbent assay
FISH: fluorescence in situ hybridization
hnRNP: heterogeneous nuclear ribonucleoprotein
IC50: half maximal inhibitory concentration
LLC: Lewis lung carcinoma
NSCLC: non-small cell lung cancer
OGA: O-GlcNAcase
OGT: O-linked N-acetylglucosamine transferase
qRT‒PCR: Quantitative reverse transcriptase polymerase chain reaction
RMSD: root mean square deviation
RMSF: root mean square fluctuation
RIP: RNA immunoprecipitation
SASA: solvent-accessible surface area
SRA: sequence read archive
TCM: traditional Chinese medicine
