# Supplementary material for: Mannose Inhibits NSCLC Growth and Inflammatory Microenvironment by Regulating Gut Microbiota and Targeting OGT/hnRNP R/JUN/IL-8 Axis
Source: Int J Biol Sci. 2025 Jan 27;21(4):1566–84. doi: 10.7150/ijbs.107256 (PMC11844275; doi:10.7150/ijbs.107256)
Supplement: Supplementary file 1 — Supplementary figures and tables. [file ijbsv21p1566s1.zip › Supplementary legends.pdf]

Supplementary Figure 1: IC50 value of mannose in H1299, A549, SK-MES-1, and PC-9 cells.
Supplementary Figure 2: (A) Ecological network of identified differential bacteria in the control and Huaier groups. (B) Combined KEGG pathway enrichment analysis of the metagenomic sequencing and blood metabolomics data. (C) Combined KEGG pathway enrichment analysis of metagenomic sequencing and fecal metabolomics data.
Supplementary Figure 3: (A) Expression level of hnRNP R in lung cancer tissues and adjacent nontumor tissues in the TCGA database. (B) Expression level of hnRNP R in different stages of lung cancer according to the TCGA database. (C) Overall survival analysis of lung cancer patients with different hnRNP R expression levels in the TCGA database. (D) Disease-free survival analysis of lung cancer patients with different hnRNP R expression levels on the basis of data from the TCGA database. (E) Correlation between hnRNP R and JUN expression in lung cancer tissues. (F) Correlation between hnRNP R and JUN expression in adjacent nontumor tissues.

Supplementary Table 1: Shotgun metagenomic sequencing.
Supplementary Table 2: Identified differential blood metabolites neg.
Supplementary Table 3: Identified differential blood metabolites.
Supplementary Table 4: Identified differential fecal metabolites neg.
Supplementary Table 5: Differential fecal metabolites identified.
Supplementary Table 6: Identified differentially expressed genes.
Supplementary Table 7: Coimmunoprecipitation-mass spectrometry data.
