# Supplementary figures and images for: Mannose Inhibits NSCLC Growth and Inflammatory Microenvironment by Regulating Gut Microbiota and Targeting OGT/hnRNP R/JUN/IL-8 Axis
Source: Int J Biol Sci. 2025 Jan 27;21(4):1566–84. doi: 10.7150/ijbs.107256 (PMC11844275; doi:10.7150/ijbs.107256)

Supplementary Figure 1

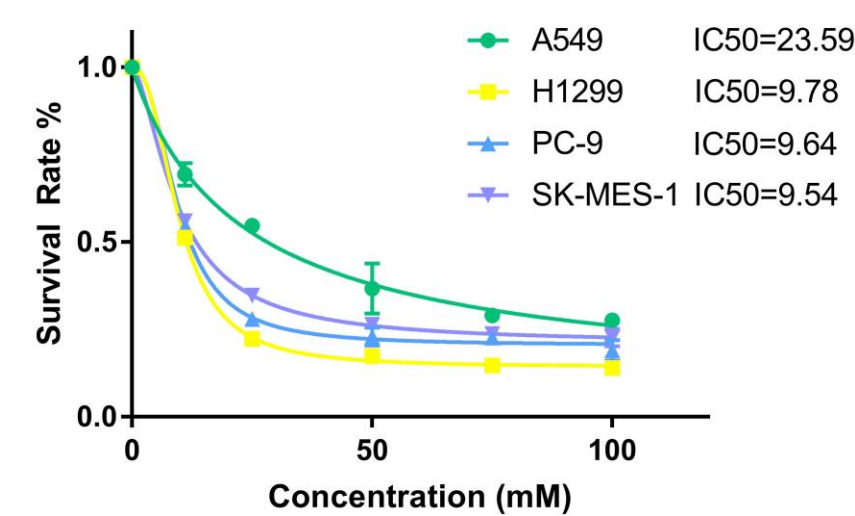

Supplementary Figure 2

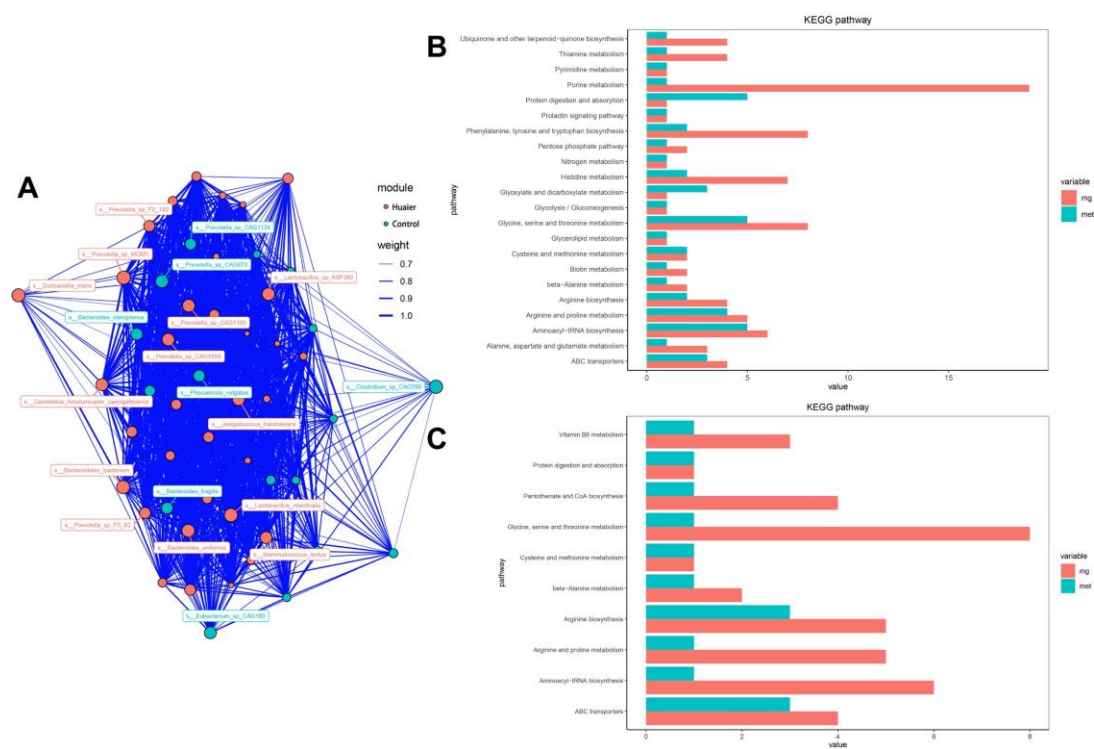

Supplementary Figure 3

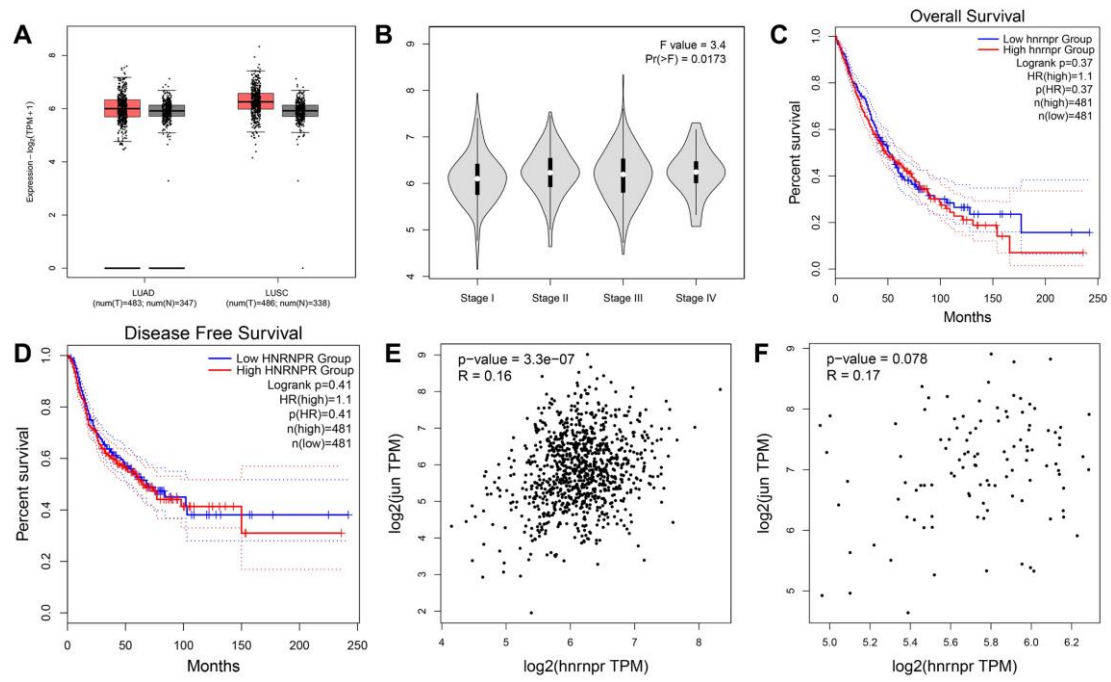

Supplement: Supplementary file 1 — Supplementary figures and tables. [file ijbsv21p1566s1.zip › Supplementary figures.pdf]
